# The diagnostic accuracy of direct agglutination test for serodiagnosis of human visceral leishmaniasis: a systematic review with meta-analysis

**DOI:** 10.1186/s12879-020-05558-7

**Published:** 2020-12-11

**Authors:** Mehdi Mohebali, Hossein Keshavarz, Sedigheh Shirmohammad, Behnaz Akhoundi, Alireza Borjian, Gholamreza Hassanpour, Setareh Mamishi, Shima Mahmoudi

**Affiliations:** 1 Department of Medical Parasitology and Mycology, Tehran University of Medical Sciences, School of Public Health, Tehran, Iran; 2 Center for Research of Endemic Parasites of Iran (CREPI), Tehran University of Medical Sciences, Tehran, Iran; 3 Pediatric Infectious Diseases Research Center, Tehran University of Medical Sciences, No. 62, Dr. Gharib St., Tehran, Iran

**Keywords:** Direct agglutination test, Visceral leishmaniasis, Diagnostic accuracy, Human, Systematic review, Meta- analysis

## Abstract

**Background:**

Direct agglutination test (DAT) as a simple, accurate and reliable method, has been widely used for serodiagnosis of visceral leishmaniasis (VL) during the last three decades. The present study is a systematic review and meta-analysis to evaluate the diagnostic accuracy of DAT for serodiagnosis of human VL.

**Methods:**

Electronic databases, including MEDLINE (via PubMed), SCOPUS, Web of Science, SID and Mag Iran (two Persian scientific search engines) were searched from December 2004 to April 2019. We determined the pooled sensitivity and specificity rates of DAT for the diagnosis of human VL, calculated positive and negative likelihood ratios (LR+ and LR-), and constructed summary receiver operating characteristic (ROC) curves parameters across the eligible studies.

**Results:**

Of the 2928 records identified in the mentioned electronic databases and after examining reference lists of articles, 24 articles met inclusion criteria and were enrolled in the systematic review and out of them 20 records qualified for meta-analysis. The pooled sensitivity and specificity rates of DAT was 96% [95% CI, 92–98] and 95% [CI95% 86–99], respectively. The likelihood ratio of a positive test (LR+) was found to be 21 [CI95%, 6.6–66.5] and the likelihood ratio of a negative test (LR−) was found to be 0.04 [(CI95%, 0.02–0.08]. The combined estimate of the diagnostic odds ratio for DAT was high [467 (CI95%, 114–1912]). We found that the summary receiver operating characteristic curve (SROC) is positioned near the upper left corner of the curve and the area under curve (AUC) was 0.98 (95% CI, 0.97 to 0.99).

**Conclusion:**

Referring to our analysis, we determined that DAT can be considered as a valuable tool for the serodiagnosis of human VL with high sensitivity and specificity. As DAT is a simple, accurate and efficient serological test, it can be recommended for serodiagnosis of human VL particularly in endemic areas.

## Background

Visceral leishmaniasis (VL) is one of the most important neglected tropical diseases that is caused by *Leishmania donovani* and *L. infantum*/*chagasi* in both humans and canines [1–3]. The morbidity and mortality due to VL are estimated at 200,000 to 400,000 new cases a year and approximately 20,000 to 40,000 deaths occur annually [4]. More than 90% of all VL cases occur in 6 countries including India, Bangladesh, Brazil, Ethiopia, Sudan, and South Sudan [5].

Since the fatality rate of VL is high and it can reach 100% when not treated properly, a diagnostic test with both high validity and reproducibility rates is required [1, 6].

Early diagnosis and treatment is the first step for elimination of anthroponotic form of VL caused by *L.donovani* because post kala-azar dermal leishmaniasis (PKDL) cases are potential reservoir hosts in the endemic areas of Indian form of kala-azar, while in Mediterranen form of kala-azar caused by *L.infantum/chagasi*, infected canines are potential role as reservoir hosts of the disease [1, 4].

Treatment of VL cases should be recommended after confirmation of the disease.

Ambisome® in VL is the first line treatment in pregnancy, for patients whose condition is severe and HIV co-infected patients [1]. A follow–up visit 6 months after the end of treatment is recommended to make sure the treatment was successful. The gold standard for diagnosing VL is mainly parasitological examinations including the demonstration of parasites by microscopic examination of splenic or bone-marrow aspiration [4, 6].

Sample preparation for parasitological examinations of VL is highly invasive. In spite of the high specificity of parasitological examinations, the sensitivity of these methods depends on the type and preparation of samples [7]. Moreover, the accuracy and precision of parasitological examinations is also subject to the experience of the laboratory microscopist. Molecular methods including polymerase chain reaction (PCR)-based methods have been developed and assessed for human and canine VL using various target genes and different clinical specimens [8]. The pooled sensitivity and specificity of PCR for detection of infections caused by *L.infantum*/*chagasi* on human peripheral blood samples were reported as 93.1 and 95.6%, respectively [7, 8]. The main limitation of PCR assays is the lack of standardization due to the large numbers of different administrative protocols [7]. In addition, the specificity of the molecular methods varied significantly in different studies in the literature due to the fact that in some studies parasitological methods are not able to identify true-positive patients [7]. Moreover, these methods require specific and expensive equipment and materials and also the performance of molecular methods particularly in remote areas has some limitations.

Several serological tests are used for serodiagnosis of VL, including the indirect fluorescent antibody test (IFAT), the enzyme linked immunosorbent assay (ELISA), the latex agglutination test (LAT), immunoblotting, the direct agglutination test (DAT) and the rK39 rapid diagnostic test. Among which, DAT and rK39 are simple and do not require sophisticated equipment; thus, they are usually used in field studies as well as in laboratories and seems to be a better screening test particularly in symptomatic VL.

One of the major drawbacks of serological tests is that they are not able to detect relapses among VL cases because anti-*Leishmania* antibodies remain in the body for a long time after clinical cure [9, 10] and many people who live in endemic areas have anti-*Leishmania* antibody titers due to high exposure to *Leishmania* parasites. In the previous meta-analysis of the diagnostic performance of the DAT for VL from January 1986 to December 2004, the pooled sensitivity and specificity rates were calculated as 94.8 and 86%, respectively [11]. This study aimed to update the diagnostic accuracy of the DAT for human VL diagnosis from December 2004 to April 2019.

## Methods

### Study design

In this study, all the studies related to diagnostic performance of DAT during 2004–2019 were systematically searched.

Selection of articles was made independently by two reviewers (SSH and AB) and the possible discrepancies were solved by consensus after discussion.

This systematic review with meta-analysis was conducted as per PRISMA (Preferred Reporting Items for Systematic Reviews and Meta-Analysis) guidelines [12].

### Search strategy

Electronic databases, including MEDLINE (via PubMed), SCOPUS, Web of Science and two Persian scientific search engines “Scientific Information Database” (www.sid.ir) and “Mag Iran” (www.magiran.com) were searched systematically with various combinations of the following scientific keywords: “Visceral leishmaniasis”, “*Leishmania infantum*”, “*Leishmania donovani*” “DAT”, “Direct Agglutination Test*”, “*Parasitology”, “Microscopy”, “Specificity” and “Sensitivity” using “OR” and/or “AND”. The references of included articles were additionally screened manually. Abstracts of articles which published in congresses were not explored.

### Case definition

Patients in VL-endemic countries with pathogonomonic signs and symtomes such as fever > 2 weeks, hepatosplenomegaly, progress weakness, anaemia or pancytopenia, and lymphadenopathy with confirmation of following testsparticularly in VL endemic areas were considered as VL cases.

#### Parasitological diagnosis

(a)

Microscopic findings of bone marrow aspirate, lymph node aspirate, and/or spleen aspirate for detection of amastigote form of *Leishmania* spp. using direct semi-quantified microscopic examination of smears.

(b)

Culture of bone marrow, lymph node, and/or spleen aspirations in Novy–MacNeal–Nicolle (NNN) media. Demonstration of at least one amastigote upon microscopic examination of tissue smears or one promastigote in culture is sufficient for the diagnosis.

#### Serological tests

DAT was performed on each sample in standardized conditions, and each test was read by two readers who were blind to other test results.

### Study selection and data extraction

Titles and abstracts of all articles were screened by one reviewer, and eligibility of the screened articles was assessed by two independent investigators using the following criteria.

Inclusion criteria were: a) have a full description of accuracy of DAT as a diagnostic test for the detection of VL in patients. b) Articles were published from December 2004 to April 2019. c) including both DAT test and parasitological examinations (direct demonstration or culture) as the diagnostic method for VL. c) Cases with no previous history of VL were included.

Exclusion criteria were: a) insufficient primary and/ data for calculating of sensitivity and specificity), b) unavailable full text article, and c) written in a language other than English or Persian.

Eligibility of all explored papers was assessed by three reviewers. All possible discrepancies among studies were resolved by discussion and consensus. The extracted data were gathered using a pre-designed form containing all the descriptive variables and test results. The following information was extracted: authors, year and country in which the study was carried out, diagnostic methods applied, reference test used, cut off, characteristics of the participants, quality of the study and sample size.

### Assessment of study quality

We assessed the quality of studies using the Quality Assessment of Studies of Diagnostic Accuracy Approach-QUADAS [13].

### Data synthesis and statistical analysis

All meta-analysis methods were performed using STATA (Release 12. statistical software. College Station, Texas: STATA Corp LP). Sensitivity and specificity were calculated using the available extracted data. To calculate sensitivity and specificity values for the tests, we extracted raw data from primary studies to calculate true positives, false positives, true negatives, and false negatives.

In the cases that the numbers of true positive, false negative, true negative, and false positive observations were not available, we derived the numbers from the marginal totals and the reported sensitivity and specificity.

Meta-analyses were accomplished by using random-effects inverse-variance weights. Results of the meta-analysis were illustrated by a forest plot diagram, which demonstrated the accuracy estimates and their relevant 95% confidence interval (CI).

The Cochran’s *Q* and *I*^*2*^ statistics were used for assessment of the between-study inconsistency and heterogeneity, respectively. The *I*^*2*^ values of 25, 50, and 75% were representatives of low, moderate and high heterogeneity, respectively. Publication bias was evaluated through the Deeks’ funnel plots [14].

## Results

**Fig. 1:**
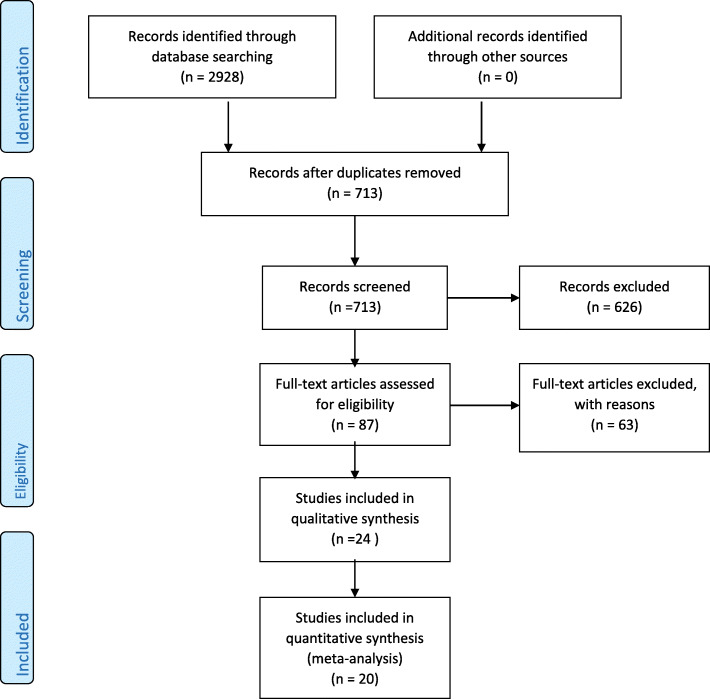
Flowchart of study selection

From the literature searches, we identified 2928 primary citations from electronic databases and through articles’ reference lists. Study selection flow is shown in Fig. 1.

Among all records, 24 articles met inclusion criteria and included into the systematic review. The characteristics of the included studies are summarized in Table 1.

**Table 1:** The characteristics of the included studies

Author	Country	Year	Sample size	Diagnostic method	Cut off	Prasite	DAT + Parasitology+	DAT + Parasitology-	DAT-Parasitology +	DAT-Parasitology -	QUADAS score
Bimal [15]	India	2005	799	Bone marrow and spleen aspiration	≥1:800	*L.donovani*	99	0	9	691	12
Silva [16]	Brazil	2005	16	Bone marrow aspiration	≥1:800	*L.donovani*	16	0	0	0	11
Sundar [17]	India	2006	329	Spleen aspiration	≥1:3200	*ND*	227	3	5	94	13
Sundar [18]^e^	India	2006	508	Spleen aspiration	≥1:1600	*ND*	144	54	6	304	13
Sundar [18]^f^	India	2006	508	Spleen aspiration	≥1:1600	*ND*	146	45	4	313	13
Kumar [18]	India	2006	548	Bone marrow and spleen aspiration	≥1:800	*L.donovani*	63	4	17	464	10
El Mutasim [19]	Sudan	2004–2005	302	Lymph nodes, bonemarrow or spleen aspiration	≥1:3200	*L.donovani*	92	16	7	187	13
Gavgani [20]	Iran	2007	12	Bone marrow aspiration	≥1:3200	*L.infantum*	12	0	0	0	9
Mansour [21]	Sudan	2004–2006	174	Lymph node aspirate	≥1:3200	*L.donovani*	24	1	1	148	11
Teran-Angel [22]	Venezuela	2007	54	Bone marrow or spleen aspiration	> 1:1:600	*L.donovani*	26	0	0	28	10
Taran [23]	Iran	2004–2006	15	Bone marrow aspiration	≥1:3200	*L.infantum*	12	3	0	0	12
Sinha [24] ^a^	India	2008	18	Spleen or bone marrow aspiration	≥1:800	*L.donovani*	6	2	0	10	11
Sinha [24] ^b^	India	2008	20	Spleen or bone marrow aspiration	≥1:800	*L.donovani*	10	0	0	10	11
Kilic [25]	Turkey	2008	59	Bone marrow aspiration - culture	≥1:1600	*L.donovani*	24	4	0	31	12
Mandal [26]	India	2005–2006	57	Spleen and bone marrow aspiration	≥1:800	*L.donovani*	16	1	0	40	10
Ghatei [27]	Iran	2005–2009	90	Bone marrow or spleen aspiration	≥1:3200	*L.infantum*	27	0	2	61	12
Hailu [28]	Ethiopia	2006	137	Spleen or lymph node aspiration - cultures	≥1:1600	*ND*	41	20	43	33	10
Mansour [29]	Sudan	2004–2005	322	Lymph node aspirate	≥1:3200	*L.donovani*	114	17	11	180	11
Ter Horst [30]	Ethiopia	2006–2007	699	Spleen aspiration	≥1:3200	*L.donovani*	144	338	9	208	10
Gani [31]	Iraq	2004–2006	57	Bone marrow aspiration	≥1:800	*ND*	10	0	47	0	11
Topno [32]	India	2005	355	Bone marrow or spleen aspiration	≥1:800	*L.donovani*	5	34	0	316	12
Abass [33]	Sudan	2011	183	Lymph node aspiration	≥1:3200	*L.donovani*	100	6	0	77	11
El. Moamly [34] ^c^	Saudi Arabia	2004–2006	33	Bone marrow aspiration - culture	≥1:1600	*L.donovani*	9	1	1	22	12
El. Moamly [34] ^d^	Saudi Arabia	2004–2006	65	Bone marrow aspiration - culture	≥1:1600	*L.donovani*	24	2	1	38	12
Machado [35]	Brazil	2004–2007	356	Bone marrow smear	≥1:3200	*L.infantum*	192	41	21	102	8
Junior [36]	Brazil	2015	7	Bone marrow aspiration	≥1:3200	*L.infantum*	3	4	0	0	10
Osman [37]^g^	Sudan	2016	135	Lymph node aspirate	≥1:3200	*L.donovani*	92	0	1	42	11
Osman [37]^h^	Sudan	2016	141	Lymph node aspirate	≥1:3200	*L.donovani*	95	0	4	42	11

^a^Coinfection of VL in HIV+patients

^b^VL in immunocompatent patients

^c^Sudanese VL patients

^d^Asian VL patients

^e^Freezed Dried DAT(FD) antigen

^f^Aqueous DAT antigen (AQ)

^g^Freezed Dried (FD) DAT antigen

^h^Liquid (LQ) DAT antigen

The reference method for diagnosis of VL in all studies was a positive result on microscopic examination and/or culture of lymph node, bone marrow, or spleen aspirates [25, 28, 34].

Some studies performed DAT using both freeze-dried (FD) and aqueous (AQ) antigen [18, 37]. No significant difference in the sensitivity of the DAT (FD and AQ) was found. In one study [37], relatively higher sensitivity (99%) was recorded for the LQ-DAT than for the FD-DAT (96%) that might be due to the use of the endemic autochthonous *Leishmania donovani* isolate as the antigen.

In overall, studies had a wide geographical distribution and were carried out in different countries including Brazil [16, 35, 36], Ethiopia [28, 30], India [15, 17, 18, 24, 26, 32], Iraq [31], Iran [20, 23, 27], Saudi Arabia [34], Sudan [19, 21, 29, 33, 37], Turkey [25] and Venezuela [22].

The study quality analysis as assessed by QUADAS tool showed that 22 out of 24 studies (92%) met more than seven criteria (Table 1).

Among 24 studies, 20 records were qualified for meta-analysis. All studies except 2 reports (one cohort [32] and one case-control [34] were cross sectional studies evaluating the diagnostic accuracy of DAT and meta-analysis was performed on all cross sectional studies evaluating the diagnostic accuracy of DAT. Three studies were excluded because there was zero cell and sensitivity and/or specificity cannot be calculated. Cut off dilution for a positive test on DAT reported to vary between 1∶800 to 1∶3200 (Table 2).

**Table 2:** Diagnostic accuracy of DAT for diagnosis of VL based on different cut off values and *Leishmania* species

Parameter	*Leishmania* species		Cut off			
	*L.donovani*	*L.infantum*	All	1:800	1:1600	1:3200
Sensitivity	96 [92–98]	92 [83–96]	96 [92–98]	95 [66–100]	97 [94–99]	96 [93–97]
Specificity	98 [93–99]	98 [15–100]	95 [86–99]	99 [93–100]	87 [83–90]	93 [73–99]
Positive Liklihood Ratio	48.6 [13.6–174]	40 [0.2–8793]	21 [6.6–66.5]	107 [12.7–901]	7.5 [5.6–10]	14.5 [3.1–66.6]
Negative Liklihood Ratio	0.04 [0.02–0.08]	0.09 [0.04–0.18]	0.04 [0.02–0.08]	0.05 [0.01–0.44]	0.03 [0.01–0.07]	0.05 [0.03–0.08]
Diagnostic Odds Ratio	1228 [280–5393]	467 [1–147,442]	467 [114–1912]	2206 [155–31,482]	236 [83–674]	309 [56–1721]

**Fig. 2:**
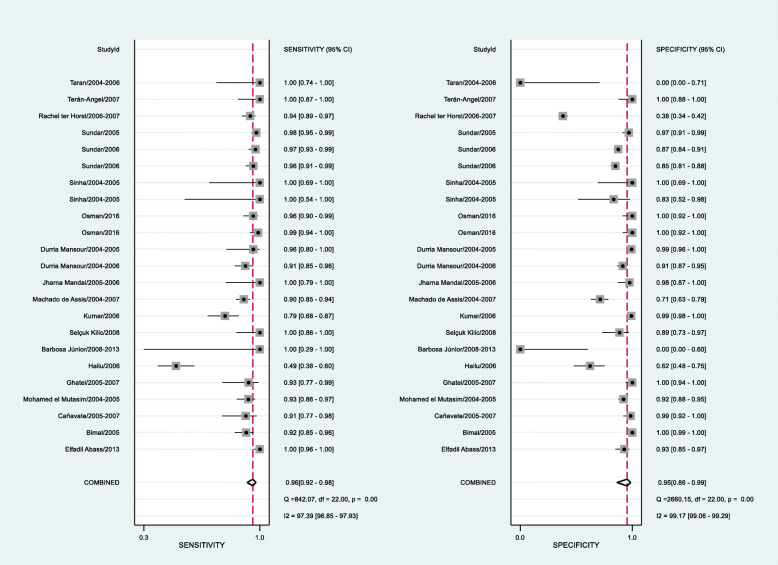
Forest plot showing the sensitivity and specificity of DAT in the diagnosis of VL

**Fig. 3:**
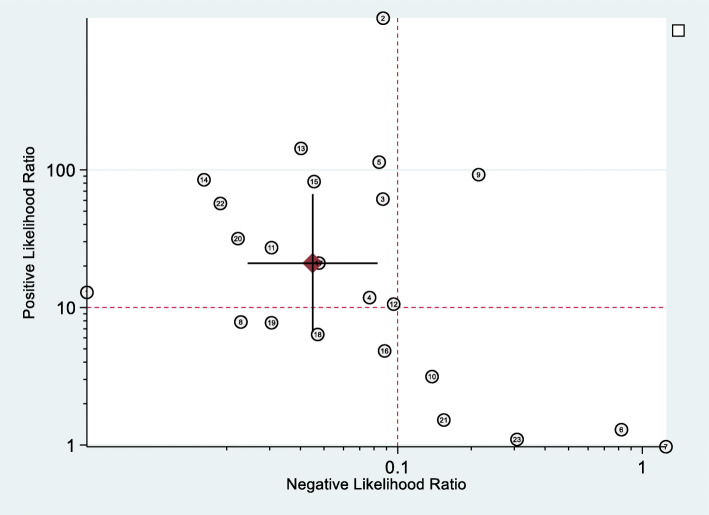
Positive and negative likelihood ratio of the included studies

Figures 1 shows the results of individual and combined sensitivity and specificity estimates of the DAT test for diagnosis of VL. The pooled sensitivity of the included studies evaluating the DAT was 96% [CI95% 92–98] and the pooled specificity was 95% [CI95% 86–99] (Fig. 2). The likelihood ratio of a positive test (LR+) was found to be 21 [CI95% 6.6–66.5] and the likelihood ratio of a negative test (LR−) was found to be 0.04 [CI95% − 0.02–0.08] (Fig. 3). Moreover, the combined estimate of the diagnostic odds ratio for DAT was high (467 [114–1912]).

Among studies which considered DAT cut off value of 1∶800, the pooled sensitivity and specificity was 95% [CI95% 66–100] and 99% [CI95% 93–100], respectively. With considering this cut off value, the LR+ was found to be 107 [CI95% 12.7–901] that was more higher than LR+ obtained from analysis of studies with cut off value of 1:1600 and 1:3200. Although the pooled sensitivity of DAT with cut off value of 1:1600 and 1:3200 was remained stable (97 and 96%, respectively), the pooled specificity was declined to 87% [CI95%83–90] with cut off value of 1:1600 and 93% [CI95% 73–99] with cut off value of 1:3200, respectively.

The pooled sensitivity and specificity of DAT for serodiagnosis of *L. donovani* was 96% [CI95% 92–98] and 98% [CI95% 93–99], respectively; while it was 92% [CI95% 83–96] and 98% [CI95% 15–100] for serodiagnosis of *L. infantum*, respectively (Table 2).

**Fig. 4:**
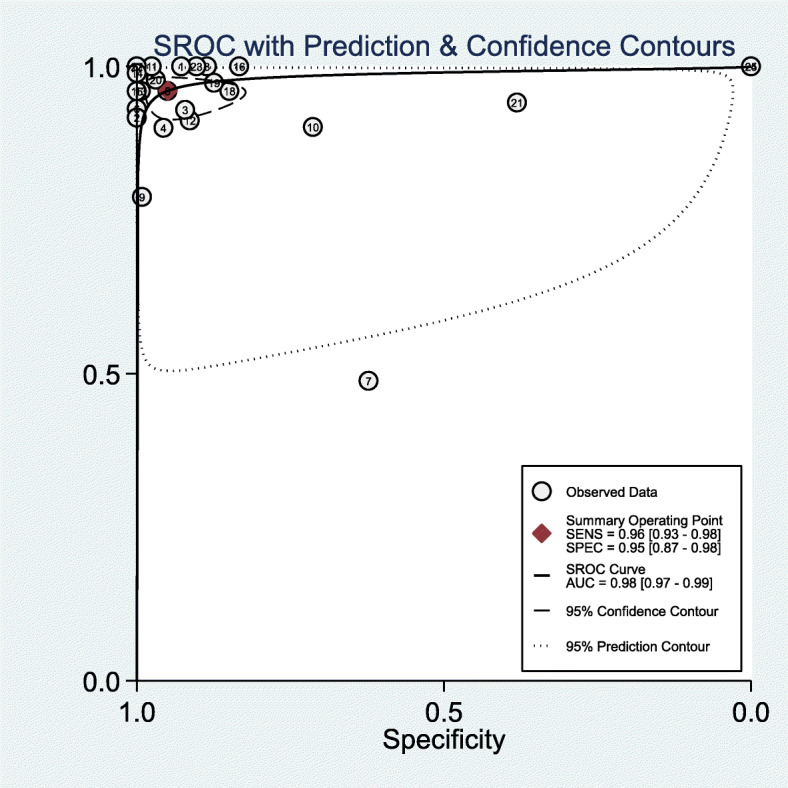
Summary receiver operating characteristic (SROC) curve for assessment of the diagnostic accuracy of DAT for the diagnosis of VL

We found that the summary receiver operating characteristic curve (SROC) is positioned near the upper left corner of the curve and the area under curve (AUC) was 0.98 (95% CI, 0.97 to 0.99) (Fig. 4).

**Fig. 5:**
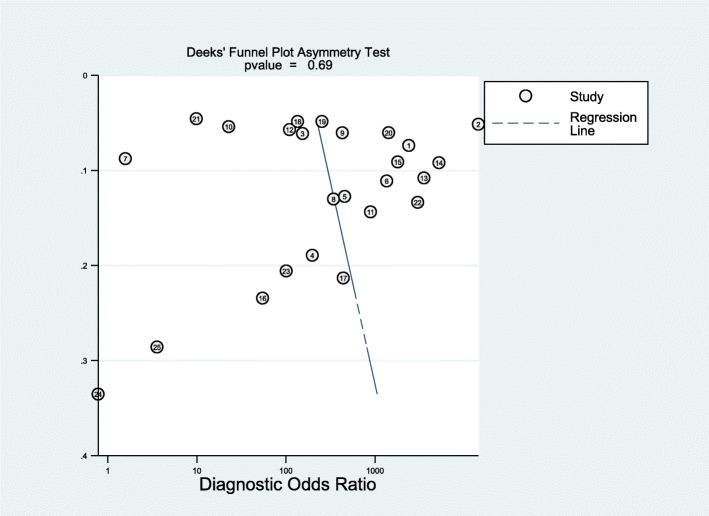
Deeks’ funnel plots for publication bias

The Deeks’ funnel plots for publication bias also showed no asymmetry (Fig. 5). The evaluation of publication bias showed no potential for publication bias (*p* = 0.69).

## Discussion

The first line regimen for primary VL treatment is pentavalent antimonial compounds such as meglumine antimoniate (Glucantime®) and Sodium stibogluconate(Pentostam®) which can be used as a monotherapy (administered as intramuscular injections of 20 mg/ kg/day for 28–30 days) or in combination with cryotherapy or other drugs such as paramomycin [1–3]. Other drugs such as amphotericin B deoxycholate, Liposomal amphotericin B (Ambisome®) and miltefosine (administrated orally) may be used for the treatment of VL particularly in patients with clinical or laboratory resistance to pentavalent drugs as well as contraindications caused by these drugs [1].

Until 1990, VL diagnosis was in need of parasitological confirmation including microscopy or culture of the spleen, bone-marrow, lymph nodes and sometimes peripheral blood specimens. The invasiveness and sometimes fatal complications particularly associated with splenic aspiration brought about the development of simple and accurate serological tests such as DAT [38]. Although the IFAT and ELISA are two important serological methods for diagnosis of human VL they require specific materials and equipment [1, 33]. The rK39 dipstick is simpler than DAT for screening of human VL particularly in symptomatic cases. The rK39-based tests are easy to perform, quick, cheap and give reproducible results and can therefore be used for early diagnosis of VL at both peripheral and central levels of public health centers Studies comparing the rK39 strip test and DAT found a similar sensitivity (94%) and specificity (89%); however, the DAT showed a slightly higher specificity [39].

On the other hand, rK39 strip tests have certain limitations because anti-*Leishmania* antibodies can persist for months in patients even after recovery, moreover, it has low specificity in sub-clinical and asymptomatic forms of *L.donovani* or *L.inantum/chagasi* infections, particularly among the Sudanese population [33, 37]. A recent introduced assay based on the detection of antibodies to the rk28 fusion protein showed a very promising sensitivity and specificity (96 and 98%, respectively) of ELISA to detect anti-*Leishmania* antibodies in sera among VL patients. In addition, The rK26, A2-ELISA and rKE16 dipstick recombinant antigens from amastigote forms of *L.infantum* or *L.donovani* showed desirable results [40–42]. Among the available serological tests for the diagnosis of VL, DAT is a simple, highly specific and sensitive, reliable and cost-effective test that can be used in field as well as laboratory studies [43–47]. DAT as a semi-quantitative serological test has been used for the serodiagnosis and seroepidemiological studies of VL in both humans and animal reservoir hosts during the last 4 decades. According to our knowledge, this is the second systematic review and meta-analysis about the diagnostic accuracy of DAT for diagnosis of VL. Chappuis et al. included 30 relevant studies that evaluated DAT from January 1986 to December 2004 [11] and reported the pooled sensitivities and specificities of 94.8 and 86%, respectively. In the present study, 24 eligible studies from April 2004 to December 2019 were evaluated using DAT for the diagnosis of VL in immunocompetent patients by the systematic review. Our meta-analysis showed DAT is still a validated serodiagnostic test with high pooled sensitivity of 95% [CI95%,66–100], 97% [CI95%,94–99] and 96% [CI95%, 93–97], respectively while 1:800, 1:1600, and1:3200 cut-off titer were considered. Higher pooled specificity 99% (95% CI, 93–100) was found at a 1:800 cut-off titer.

Specific *Leishmania* antibodies at a titer of 1:800 showed VL infection [33] whereas *Leishmania* antibodies at a titer of 1:3200 with pathogonomonic clinical signs such as hepatosplenomegaly, dromedary fever, anemia and progress weakness reflected active VL [43].

The high diagnostic accuracy of DAT using different samples including serum, plasma or even urine samples has been reported [11, 48].

According to the of the previous meta-analysis comparing the diagnostic accuracy of DAT and rK39 strip test, DAT showed 1% more sensitivity and 2% more specificity than rK39 strip test.

Although we did not have sufficient information regarding human immunodeficiency viruses status of the cases, it has been reported that DAT has an acceptable sensitivity in the diagnosis of VL in HIV-positive patients [49]. Although sensitivity of DAT in the majority of studies was more than 90% some heterogeneity in the sensitivity of the tests might be related to the geographical location of the study, differences in antibody concentrations, and immune or nutritional status of the patient [50]. In the current study, it was not possible to evaluate the test performance according to these factors.

Although early diagnosis and appropriate treatment is crucial for controling of the anthroponotic form of VL (i.e. Indian and African forms of VL), control of zoonotic VL is highly difficult and the current control strategies for zoonotic VL rely on animal reservoir hosts and phlebotomine vectors [1], the use of insecticide-impregnated materials to prevent insect bites, and active case detection with appropriate treatment to decrease the mortally rate of VL [45].

VL is highly fatal in the absence of appropriate anti-*Leishmania* drugs. Although pentavalent antimonial compounds are introduced as the drugs of choice for the treatment of VL, intramuscular or painful intravenous injections with high toxicity and high price consider. However, anti-leishmanial drugs are usually expensive and have significant toxicity. Since the clinical manifestations of VL have low specificity and are not completely pathognomonic, performing of confirmatory tests to recognize the VL patients needing treatment are highly required. These tests must not only be sensitive, but they also need to be specific because the current anti-*Leishmania* drugs prescribed to treat VL are highly toxic [7].

The most important limitation for the evaluation of serological tests is the absence of an appropriate gold standard test. Confirmation of VL depends on the finding of Leishman bodies of *Leishmania* sp. in samples prepared from bone marrow or spleen, lymph nodes and liver. This procedure is highly invasive, and it should be performed only in suspected cases of VL. Furthermore, the sensitivity of this method is diverse [47].

In some VL-endemic areas, DAT is used regularly for the diagnosis and sero-epidemiological studies of VL because it is simple due to its high sensitivity [33, 44].

The performance of DAT is neither *Leishmania* species-specific nor region dependent [6, 43].

Major limitations of DAT are its long incubation period to report, batch to batch variability of the antigen and cross-reactivity with *Trypanosoma cruzi* infection [43, 44]. DAT titers decline over time below the cut off (1:800), while itstill remain positive for a relatively long time (up to 5 years in more than 50% of VL cases) after the cure. Therefore, performing of this test for treatment follow up or for the diagnosis of disease relapse is not recommended [49]. To overcome the problem of long incubation time, a fast agglutination screening test (FAST) has been introduced, which uses only one serum dilution and requires only three hours of incubation [51]. FAST for the detection of *L. infantum* infection was compared with the conventional DAT in Iran indicating a sensitivity of 95.4% and specificity of 88.5% for fast DAT in comparison with conventional DAT [51].

Our study has some limitations. First, we compared pooled estimates between different published studies. So, the possibility of confounding should not be ignored. Second, the study used the data provided by published literature, and some data including sex and age for the included patients was unavailable and made subgroup analysis difficult.

## Conclusion

Based on our analysis, the use of DAT is the best choice for early detection of acute and chronic clinical forms of VL as well as the asymptomatic form of the infections in immunocompenent individuals. Further research on DAT antigen standardization in the laboratories located in endemic areas of VL is recommended.

## Data Availability

The datasets used and/or analysed during the current study available from the corresponding author on reasonable request.

## Abbreviations

DAT: Direct agglutination test
VL: Visceral leishmaniasis
FAST: Fast agglutination screening test
PCR: Polymerase chain reaction
IFAT: Indirect fluorescent antibody test
ELISA: Enzyme linked immunosorbent assay
FD: Freeze-dried
AQ: Aqueous antigen
SROC: Summary receiver operating characteristic curve
AUC: Area under curve
